# A retrospective analysis of autotransplanted teeth including an evaluation of a novel surgical technique

**DOI:** 10.1007/s00784-020-03673-y

**Published:** 2020-12-02

**Authors:** Clemens Raabe, Michael M. Bornstein, Julien Ducommun, Pedram Sendi, Thomas von Arx, Simone F. M. Janner

**Affiliations:** 1 Department of Oral Surgery & Stomatology, School of Dental Medicine, University of Bern, Freiburgstrasse 7, CH-3010 Bern, Switzerland; 2 Department of Oral Health & Medicine, University Center for Dental Medicine Basel, University of Basel, Mattenstrasse 40, CH-4058 Basel, Switzerland

**Keywords:** Autotransplantation, Apicoectomy, Root-end resection, Revascularization

## Abstract

**Objectives:**

To assess survival rates and frequency of complications for immature and mature autotransplanted teeth after at least 1 year in function.

**Materials and methods:**

All consecutive patients who had undergone tooth autotransplantation between 2000 and 2018 were invited to a clinical and radiographic follow-up examination. First, survival rates were calculated on the basis of a phone inquiry. A clinical follow-up examination allowed for the calculation of the success rate, i.e., absence of any potentially adverse clinical and radiographic findings of the autotransplanted teeth. Moreover, the effect of demographic, dental, and surgical variables on survival/success was analyzed statistically.

**Results:**

Thirty-eight teeth in 35 patients were transplanted during the study period. Three teeth in 3 patients were excluded due to missing records. All other patients were successfully contacted and interviewed by phone. Out of these 35 transplants, 32 were still in function, and 3 had been extracted, yielding a 91.4% survival probability after a median follow-up of 3.4 years. Of the 32 teeth qualifying for the success analysis, 20 (62.5%) showed absence of potentially adverse findings, while 3 (9.4%) required root canal treatment (RCT). Out of the 9 mature, root-end resected transplants, 4 exhibited ongoing pulp canal obliteration, all with a single root canal. Postoperative and potentially adverse findings or failures were found more frequently in the group of mature transplants (55.6%) than immature transplants (30.4%) and for molars (72.7%) than premolars (17.6%) or canines (25%). None of the potential predictors had a statistically significant effect on survival or success.

**Conclusion:**

Autotransplanted teeth yielded a satisfying midterm survival rate regardless of their stage of development. An additional, extraoral root-end resection of mature transplants may lead to rates of revascularization and postoperative pulp canal obliteration higher than the data reported on unmodified mature transplants.

**Clinical relevance:**

Extraoral root-end resection of mature teeth shows promising outcomes for transplants especially with a single root canal and uncomplicated root morphology.

## Introduction

Autotransplantation provides the highest grade of site-specific tissue reconstitution when replacing a missing tooth [1, 2]. Successfully transplanted teeth show unequivocal evidence of preserved pulp vitality and healthy periodontal tissues in the long term [3–5]. The major advantage of autotransplantation is the formation of a periodontal ligament (PDL) around the transplanted tooth [6]. In contrast to osseointegrated dental implants, the PDL allows the preservation and continuous growth of the alveolar ridge [2], which is particularly important in children and adolescents. Further advantages are the intact proprioception [7] and the possibility of orthodontic movements [8] of transplanted teeth. In spite of these evident biological advantages, autotransplantation is an undervalued treatment option for the replacement of missing teeth in daily routine [9]. The main reasons might be related to the inconsistent success rates reported in the literature, the demanding technique, and finally the limited availability of potential transplants with incomplete root formation.

Maintaining vitality and integrity of both periodontal and pulpal tissue directly affects the postoperative outcome. Root resorptions are found in 0–22.2% of immature and 0–100% of mature transplants [10, 11]. Pulp necrosis occurs in 0–34% of immature teeth, whereas 85–100% of teeth with mature root formation are affected [3, 12, 13]. Therefore, root canal treatment is routinely performed perioperatively for mature transplants [12, 14]. In summary, immature teeth transplanted with 2/3 to 3/4 length of root development achieve the highest success rates ranging from 61.1 to 100% [11, 15] compared to 53.3–83.8% for mature teeth [16]. This is especially relevant for patients between 9 and 12 years of age, in which immature premolars can be transplanted with documented predictability [2, 3, 13, 17, 18]. Limited evidence exists for immature third molars, which are frequently available in patients between 14.5 and 21.5 years of age [19, 20].

Recently, a novel surgical technique using extraoral apical resection for preservation of pulp vitality in mature autotransplanted teeth was presented [21]. Subsequently, two case reports have also shown successful outcomes using similar techniques [22, 23]. The promising data obtained with this novel approach is corroborated by animal studies, where root-end resection of mature transplants has yielded high revascularization rates [24–26]. Therefore, the aim of the present study was to assess the outcome of both immature and mature autotransplanted teeth after 12 or more months in function. The primary objective was to assess survival of these autotransplants. Secondary objectives included an investigation of factors influencing success rates such as mature vs. immature teeth and to document clinical and radiographic parameters associated with these.

## Material and methods

### Patient selection

For the present study, all consecutive patients who had undergone at least one tooth autotransplantation at the Department for Oral Surgery and Stomatology, University of Bern, between January 2000 and February 2018, were found by thorough electronic and hand search of the patient’s records of the department. Thereafter, they were contacted by phone. Inclusion criterion was a minimal time in function of at least 12 months. Pregnant women and subjects unable to provide informed consent as well as patients with missing or incomplete records were excluded from the study. All patients contacted were provided detailed information about the present study and were invited for a follow-up examination between November 2018 and February 2019. Informed consent was obtained from all participants by the principal investigator (PI; CR) at the beginning of the visit. The study protocol was approved by the standing ethics review board (Ethics Committee of the State of Bern, approval number 2018-01597), and the investigation was performed in accordance with the Declaration of Helsinki (2013).

### Surgery

All surgical procedures were performed under local anesthesia. The recipient site was prepared via mucoperiosteal flap elevation and osteotomy if required, in order to accommodate the transplant. The donor tooth was placed into a cell culture medium (SOS Dentobox; Miradent, Duisburg, Germany, or Dentosafe; Kaladent AG, St. Gallen, Switzerland) immediately after removal for intermediate storage during the procedure. Any root-end resection (2–4 mm) of mature teeth was performed using a sterile fissure bur under copious irrigation with Ringer’s solution. Enamel matrix derivative (EMD) was applied to the root surface upon the surgeon’s choice (Emdogain®; Straumann AG, Basel, Switzerland). Finally, the donor tooth was transplanted to the recipient site and fixated using adhesive techniques (titanium mesh or wire), sutures, or fixed orthodontic appliances where present. Medication, follow-up, and debonding protocols were chosen individually.

### Data acquisition and analysis

1. Phone call

All patients were asked to provide information about the status of the transplant upon phone inquiry, i.e., if the tooth was still in place, if any additional treatment had been performed so far, and if any symptom was present in rest or function.

2) Patient records

The records were screened with regard to the following pre- and perioperative variables: stage of eruption (erupted, partially erupted, impacted), indication for transplantation, donor and recipient site, modification of the root (i.e., root-end resection), use of EMD during surgery, and type and duration of splinting.

3) Clinical and radiographic evaluation

Patients who agreed to attend a follow-up examination were evaluated clinically and radiographically by the principal investigator (CR), who was not involved in the treatment of any of the included patients.

The following clinical parameters were assessed at the transplanted tooth and its adjacent teeth: presence of redness, swelling or fistula, periodontal probing depths (in mm) and bleeding on probing (yes/no according to Lang 1986 [27]) at both three buccal and three oral aspects, gingival recession (class I to IV according to [28], sensitivity to CO_2_-snow, pain on percussion, mobility (grades 0 to III according to Miller 1950[29], presence of occlusal contacts, and, if applicable, type of restauration.

A periapical radiograph (PA; Soredex Minray, Helsinki, Finland) using a film holder for parallel technique (XCP film holder; Dentsply Sirona, Bensheim, Germany) was taken. The images were assessed using Digora (Soredex, Helsinki, Finland) on a Flexscan monitor (Eizo, Hakusan, Japan) for the following parameters: integrity of the periodontal space, radiolucencies (periapical, intra-, para-, and periradicular) and signs of pulp canal obliteration when compared to the postoperative PA. Root formation stage prior to surgery was classified according to [20] on the preoperative PA. The stages represent the relation of the actual to the estimated final root length of the transplanted tooth. Thus, the root length was classified as R$\frac{1}{4}$, R$\frac{2}{4}$, R$\frac{3}{4},$ or complete. Teeth with complete root length were additionally allocated according to the status of the apex: open (R$\frac{4}{4}$), half-closed (A$\frac{1}{2}$), or closed apex (Ac). In the present analysis, teeth with completed root length (R$\frac{4}{4}$, A$\frac{1}{2},$ or Ac) were classified as mature and R$\frac{1}{4}$–R$\frac{3}{4}$ as immature from the perspective of the surgical protocol.

Additionally, cone beam computed tomography (CBCT) using a low-dose protocol (180° rotation at 90 kV, 5 mA, 9 s) and a limited field of view (FOV) of 4 × 4 cm (Accuitomo 170; JMorita MFG Corp., Kyoto, Japan) was performed in patients aged 16 years or older. The CBCT images were assessed for the same parameters as the 2D radiographs using the device-specific image processing software i-Dixel (JMorita MFG Corp.). In order to avoid bias by additional information from the CBCT data, PAs were assessed first. In cases of inconclusive findings, the images were evaluated by a second examiner (SJ) and discussed until consensus was reached.

### Classification

All transplanted teeth still in situ and not associated with pain upon phone inquiry were considered as surviving. Teeth associated with symptoms or having been removed were classified as failure at this first stage of the analysis.

For teeth undergoing the follow-up examination, the additional category “success” was defined by fulfilling the following criteria (adapted and modified from [30]):

- *Clinical criteria*: No pain, physiologic mobility in relation to root length (Miller grade 0–1 for R$\frac{1}{4}$ and R$\frac{2}{4}$, Miller grade 0 for R$\frac{3}{4}$–Ac), probing depths < 3.5 mm, no signs of inflammation, no pain to percussion;
- *Radiographic criteria*: intact periodontal space, no sign of periapical, intra-, para-, or periradicular radiolucency and progressing pulp canal obliteration or bone ingrowth to the pulpal chamber in transplants without root canal treatment.

Teeth presenting with one or more questionable clinical and/or radiographic criteria at the follow-up visit were classified as teeth with potentially adverse findings, i.e., allocated to the surviving teeth. Teeth deemed as a failure upon phone inquiry but with the corresponding patient not attending the visit as well as the failures with clinical examination were both considered as failures in the success analysis, assuming that most patients having had their transplant extracted would refuse a visit.

### Statistical analysis

Patient data were first analyzed descriptively. Survival probabilities were estimated using the Kaplan-Meier method. Between-group differences (mature versus immature transplants) were compared using the log-rank test. Potential factors influencing survival were analyzed using a Cox proportional hazards model.

A *p* value of 0.05 was considered as statistically significant. All statistical analyses were performed using R and RStudio (version 1.2.1335, www.r-project.org).

## Results

### Patient selection

Thirty-five (22 female, 13 male) patients underwent one (32 patients) or two (3 patients) tooth transplantations, resulting in a total of 38 autotransplantations. Three teeth in three patients had missing records and were excluded from the investigation. The median age at the time of surgery was 13 years (range 8–28 years). The most frequent indications were aplasia (*n* = 18) and failure of orthodontic extrusion of an impacted tooth (*n* = 14). Six of the donor teeth were fully erupted, seven were partially erupted, and 22 were impacted. Twenty five of the 35 transplants were immature at the time point of surgery (R$\frac{1}{4}$ (*n* = 4), R$\frac{2}{4}$ (*n* = 10), and R$\frac{3}{4}$ (*n* = 11)) and were therefore transplanted without any manipulation of the root. The 10 remaining teeth showed complete root length (R$\frac{4}{4}$ (*n* = 2) and Ac (*n* = 8)) prior to transplantation, and all underwent an intraoperative root-end resection. Thus, it was decided to regard the pairs immature/unmodified as well as mature/root-end resected as synonyms in the further analyses. Relevant patient information and the donor and recipient sites of the transplants are shown in Table 1. Treatment was performed by a total of 8 senior surgeons, i.e., trained and board-certified oral surgeons of the department. The initial postoperative healing was uneventful in all cases.

**Table 1:** Summary of demographic and perioperative variables as well as findings upon follow-up classified into success, perioperative findings, dropout, and failure

Patient			Perioperative									
Transplant, n	Age at surgery, y	Gender	Donor: stage of eruption	Recipient: indication for transplantation	Donor site (FDI)	Recipient site (FDI)	Donor: stage of root formation	Enamel-Matrix-Derivatives, y/n	Duration of surgery, min	Type of splinting	Duration of splinting, d	
Immature teeth												
1	11	f	partially erupted	Congenital tooth deformity	24	11	R3/4	y	60	TTS	14	
2	14	m	dystopic	Dystopia/Malposition	43	45	R2/4	n	300	TTS	37	
3	11	f	partially erupted	Aplasia	24	34	R3/4	n	40	TTS	35	
4	12	f	erupted	Aplasia	45	15	R3/4	n	90	TTS	25	
5	12	f	erupted	Aplasia	35	25	R3/4	n	90	TTS	25	
6	8	m	impacted	Aplasia	28	15	R3/4	n	70	Ortho	42	
7	13	f	dystopic	Dystopia/Malposition	45	45	R1/4	y	120	Ortho	85	
8	13	f	impacted	Aplasia	45	15	R2/4	n	120	WCS	33	
9	12	f	impacted	Aplasia	45	35	R2/4	n	60	TTS	58	
10	15	f	impacted	Caries	38	36	R2/4	n	90	TTS	119	
11	17	m	dystopic	Dystopia/Malposition	35	35	R3/4	n	135	TTS	101	
12	12	f	partially erupted	Aplasia	44	24	R3/4	n	n/a	WCS	n/a	
13	12	f	impacted	Aplasia	14	24	R1/4	n	50	WCS	30	
14	9	f	erupted	Aplasia	14	45	R2/4	n	n/a	Ortho	n/a	
15	17	m	dystopic	Dystopia/Malposition	35	35	R2/4	y	120	Ortho	113	
16	14	m	dystopic	Dystopia/Malposition	45	45	R3/4	y	100	TTS	58	
17	16	f	impacted	Aplasia	28	35	R1/4	n	90	TTS	n/a	
18	17	f	impacted	Aplasia	18	45	R1/4	n	120	TTS	47	
19	14	f	impacted	Dystopia/Malposition	48	46	R2/4	n	60	Suture	9	
20	13	m	partially erupted	Aplasia	35	23	R3/4	n	40	TTS	30	
21	16	m	dystopic	Dystopia/Malposition	48	47	R2/4	y	60	Suture	12	
22	17	m	impacted	Dystopia/Malposition	28	46	R1/2	y	n/a	TTS	51	
23	12	w	erupted	Aplasia	24	45	R3/4	n	n/a	TTS	48	
24	12	f	erupted	Aplasia	15	35	R2/4	n	90	TTS	19	
25	12	m	erupted	Aplasia	27	45	R3/4	n	90	TTS	35	
Mature teeth												
1	12	f	dystopic	Dystopia/Malposition	33	33	Ac	n	90	TTS	34	
2	17	f	dystopic	Dystopia/Malposition	23	23	Ac	y	60	Ortho	121	
3	22	f	impacted	Aplasia	28	45	Ac	y	150	TTS	95	
4	19	f	impacted	Dystopia/Malposition	35	35	Ac	y	150	Ortho	34	
5	27	f	impacted	Caries	18	16	Ac	y	110	TTS	26	
6	10	f	partially erupted	Dystopia/Malposition	46	46	Ac	n	130	TTS	36	
7	15	f	partially erupted	Aplasia	47	46	Ac	n	80	TTS	34	
8	28	m	impacted	Dystopia/Malposition	13	13	Ac	y	120	Ortho	163	
9	14	w	dystopic	Dystopia/Malposition	45	45	R4/4	y	n/a	TTS	28	
10	12	m	partially erupted	Aplasia	35	14	R4/4	n	50	WCS	28	
Follow-up											Classification	
Transplant, n	Follow-up, y	Time in function before failure, y	Stage of root formation	Root canal treatment, y/n	Obliteration, y/n	Max. probing depth, mm	Max. recession (mm)	Mobility	BOP, %	Sequelae	Success rate	Survival rate
Immature teeth												
1	2.1	n/a	R3/4	y	n	2.5	0.5	0	0.17	n	69.6% success	92% survival
2	1.1	n/a	R3/4	n	y	2	0	0	0.33	n		
3	10.1	n/a	Ac	n	y	3	0	0	0.33	n		
4	4.8	n/a	Ac	y	n	3	0	0	0.17	n		
5	4.8	n/a	Ac	n	y	3	0	0	0	n		
6	8.6	n/a	Ac	n	y	3.5	0	0	0.17	n		
7	2.6	n/a	R1/4	n	bi	3	0	1	0	n		
8	10.7	n/a	R3/4	n	y	3	0	0	0.17	n		
9	11.1	n/a	R3/4	n	y	2	1	0	0	n		
10	1.7	n/a	R2/4	n	y	2.5	0	1	0.33	n		
11	1.0	n/a	R3/4	n	y	3	0	0	0.17	n		
12	16.1	n/a	Ac	n	y	2.5	0	0	0	n		
13	14.3	n/a	R2/4	n	y	2.5	0	0	0.17	n		
14	15.2	n/a	R3/4	n	y	3	0	0	0.33	n		
15	1.0	n/a	R2/4	n	bi	2.5	0	0	0.17	n		
16	1.1	n/a	Ac	n	y	3	0	0	0.67	n		
17	3.3	n/a	R1/4	n	bi	3	0	0	0.5	IP	21.7% survival with sequelae	
18	2.3	n/a	R1/4	n	bi	3.5	0	0	0.67	IP		
19	12.5	n/a	R3/4	n	y	3	0	0	0.33	ICR		
20	13.6	n/a	Ac	n	y	3.5	0	0	0.67	ICR		
21	3.4	n/a	R2/4	n	y	6	1.5	1	0.33	AML		
22	n/a	n/a	n/a	n/a	n/a	n/a	n/a	n/a	n/a	n/a	exclusion	
23	n/a	n/a	n/a	n/a	n/a	n/a	n/a	n/a	n/a	n/a		
24	n/a	8.4	R2/4	n	y	n/a	n/a	n/a	n/a	IRR	8.7% failure	8% failure
25	n/a	4.1	R3/4	n	y	n/a	n/a	n/a	n/a	ICR		
Mature teeth												
1	5.3	n/a	n/a	n	y	2.5	0	0	0.17	n	44.4% success	90.0% survival
2	2.9	n/a	n/a	n	y	1.5	0	0	0	n		
3	1.3	n/a	n/a	n	y	3	0	0	0.17	n		
4	2.6	n/a	n/a	n	y	2.5	2	0	0.33	n		
5	4.3	n/a	n/a	n	n	2.5	0	0	0.5	RR	44.4% survival with sequelae	
6	2.0	n/a	n/a	n	n	9	0	0	0.67	RR		
7	2.0	n/a	n/a	y	n	8.5	1	0	0.83	RR		
8	1.5	n/a	n/a	n	n	8	1.5	0	0.83	AML		
9	n/a	n/a	n/a	n/a	n/a	n/a	n/a	n/a	n/a	n/a	exclusion	
10	n/a	4.8	n/a	n	y	n/a	n/a	n/a	n/a	ICR	11.1% failure	10.0% failure

Follow-up data is available only for patients attending the examination. Missing data (n/a) were frequent in patients with outdated surgery. Timeframes are given in (*d* = days) and (y = years). Application of EMD, RCT, or signs of obliteration are listed as (y = yes, n = no). Splinting protocols were individually chosen using titanium-trauma-splints (TTS), wire-composite-splints (WCS), existing fixed orthodontic appliances (ortho), or sutures. Sequelae are abbreviated as follows: infraposition (IP), invasive cervical resorption (ICR), apico-marginal lesion (AML), and replacement resorption (RR)

**Fig. 1:**
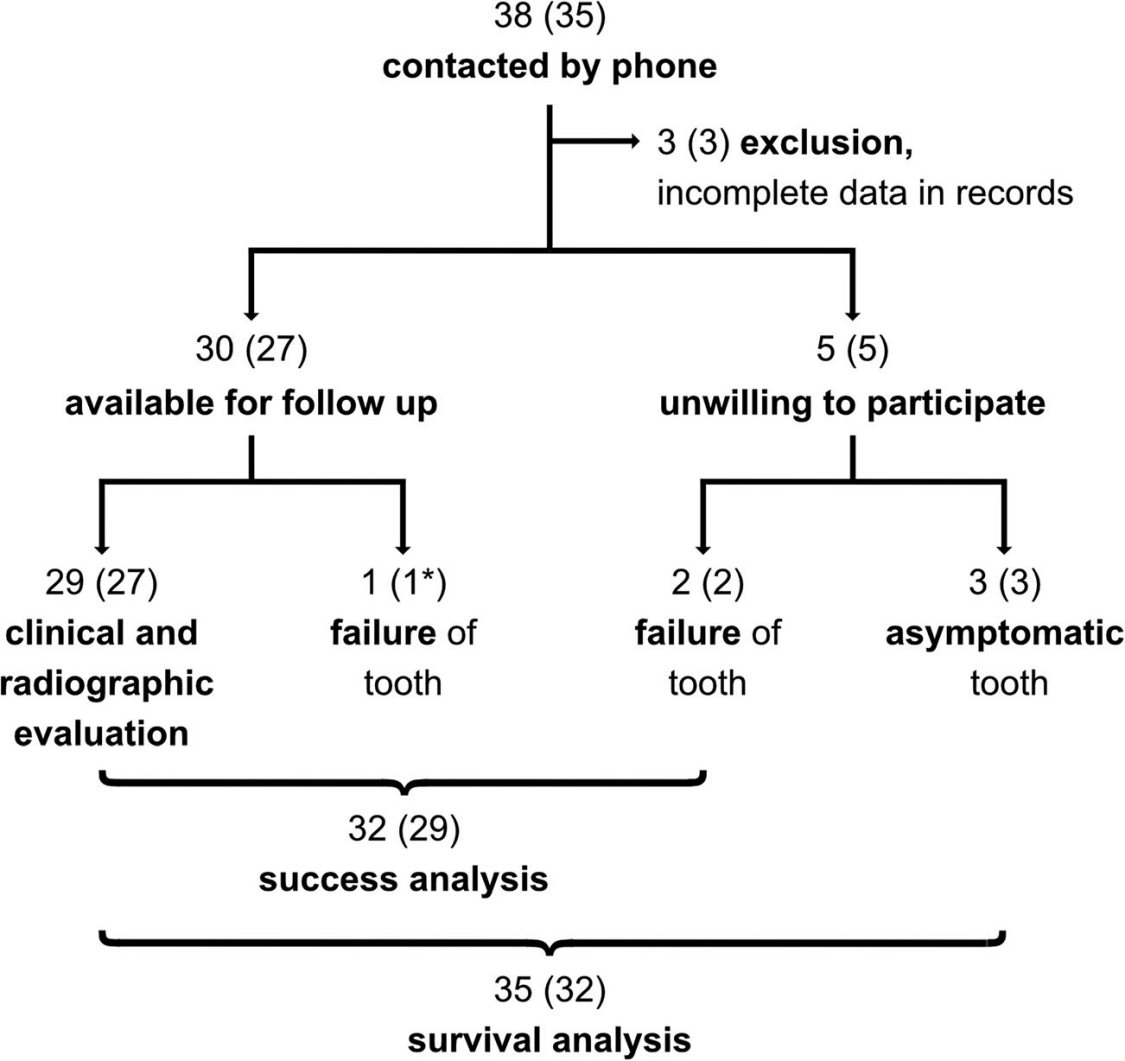
Flowchart of transplanted teeth (patients) included in the analysis. Three patients had two transplantations each and one of them had both a surviving transplant as well as a failure. The latter is marked as 1*, to avoid miscalculations. Success analysis took all failures into account (as two of them did not show up), thus excluding the 3 asymptomatic surviving teeth since no data were available

**Fig. 2:**
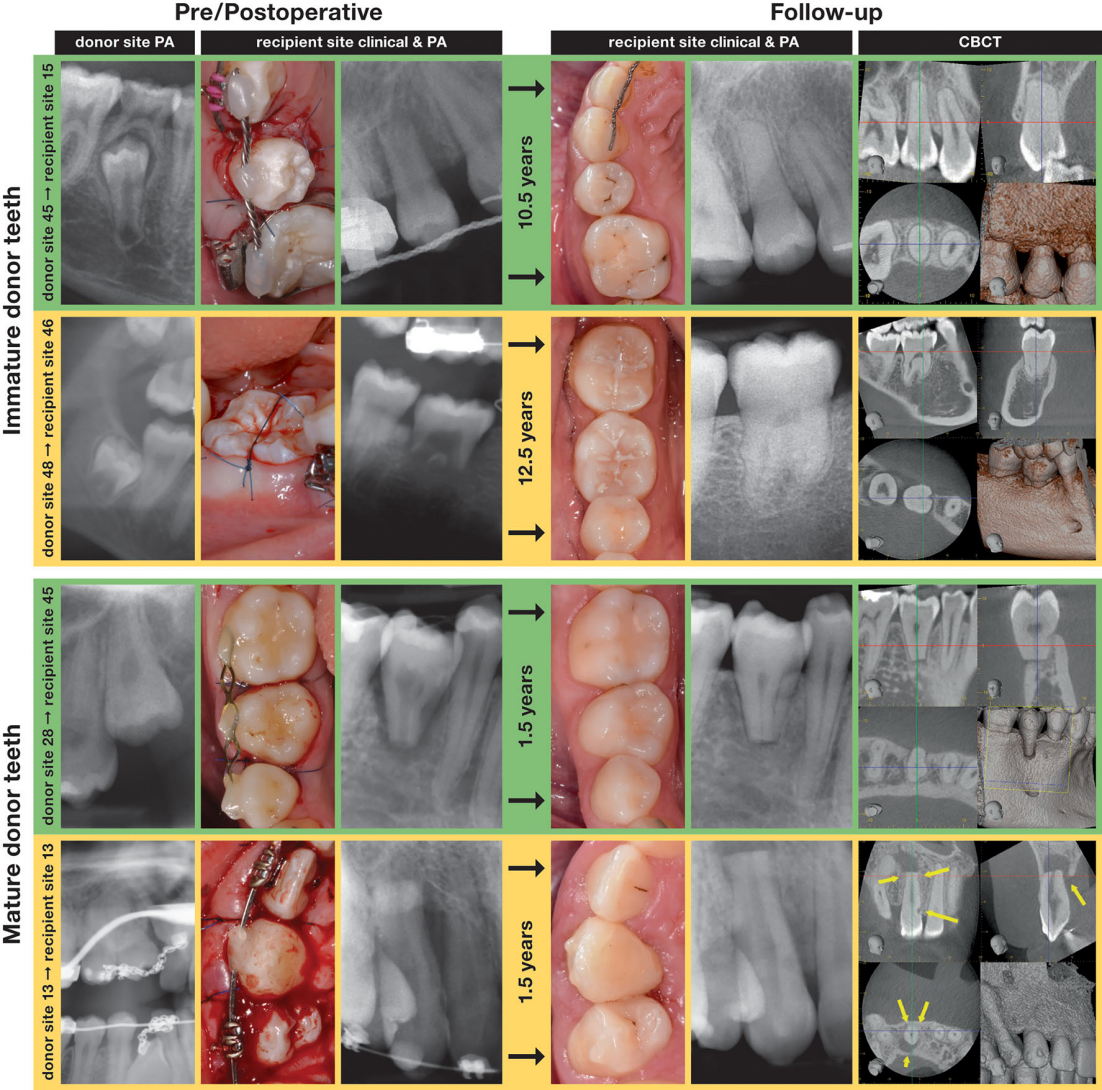
Four representative immature and mature transplants without (green) and with (yellow) postoperative potentially adverse findings. In the immature autotransplant, this finding was an intraradicular radiolucency at the level of the cementoenamel junction mesially at the transplant (i.e., invasive cervical resorption), which was only detectable with CBCT. In the mature autotransplant, the findings were discontinuous periodontal space, periapical radiolucency and an apico-marginal defect (i.e., replacement root resorption and apico-marginal lesion). Preoperative PA of the donor and recipient sites, postoperative clinical picture, and PA of the recipient site and CBCT, PA, and clinical picture at follow-up are shown

All of the 32 included patients provided information about the subjective condition of the transplant via phone; i.e., all their 35 transplanted teeth were available for survival analysis. Of these 32 included patients, 27 with 30 transplants (of which one failed) attended the follow-up examination. The two patients who reported a failure upon phone inquiry but were not willing to attend the examination are included in the success analysis (Fig. 1). Figure 2 shows representative clinical and radiographic images at surgery and at follow-up of immature and mature transplants, respectively.

### Survival analysis

**Fig. 3:**
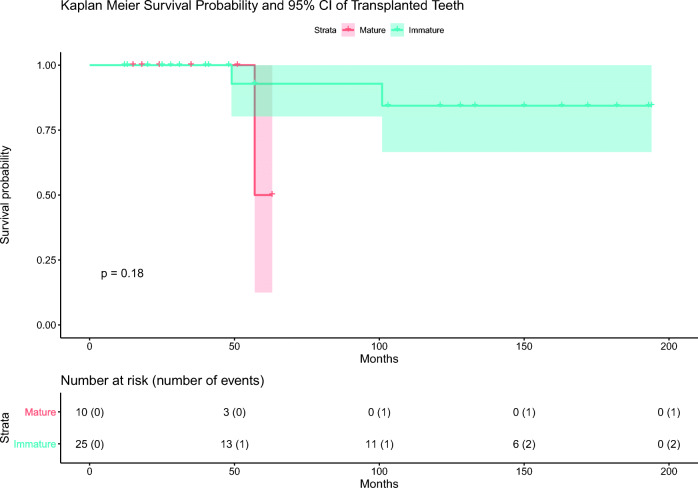
Kaplan-Meier survival probability and 95% CI of transplanted teeth

Thirty-two transplants in 30 patients are asymptomatic, and three patients report the loss of one transplant each (Table 1). For all failed transplants, the relevant data until the last follow-up visit was available in the patient records. Two of the 25 unmodified (i.e., immature) teeth were extracted, one after 4.1 years due to inflammatory root resorption (a right maxillary second premolar) and the other after 8.4 years due to combined replacement and invasive cervical root resorption (a left maxillary second molar), respectively. One of the 10 root-end resected (i.e., mature) teeth was extracted after 4.8 years due to combined replacement and invasive cervical root resorption (a left mandibular second premolar). Based on the phone inquiry, an overall survival probability of 91.4% (32 of 35 teeth) after a median observation period of 3.4 years was calculated. A more detailed analysis exhibited a 92% survival probability (23 of 25 teeth) for unmodified teeth after a median observation period of 4.8 years and 90% (9 of 10 teeth) for root-end resected teeth after a median observation period of 2.5 years. The Kaplan-Meier 5-year survival probability estimate for immature transplanted teeth was 92.9% (95% CI: 80.3–100%). For mature transplanted teeth, the corresponding 5-year survival probability estimate is 50% (95% CI: 12.5–100%; Fig. 3). The log-rank test for comparison of the Kaplan-Meier survival estimates was not statistically significant (*p* = 0.18). The calculated failure rate is approximately three times higher in the mature group as compared to the immature group, but the difference is not statistically significant (*p* = 0.52; Table 2). In a Cox proportional hazards model, none of the potential predictors (patient’s age, gender, smoking habit, number of roots, stage of root development and eruption, use of EMD, use of antibiotics, type and duration of splinting) was significantly associated with survival probabilities.

**Table 2:** Failure rate of mature and immature transplanted teeth

	Transplants (*n*)	Failures (*n*)	Person-time (months)	Failure rate (per 1000 person-months)	*p* value (immature versus mature)
Immature	25	2	2086	0.959	0.5153
Mature	10	1	346	2.890	
Total	35	3	2432	1.233	

### Follow-up examination

Twenty-seven patients with 29 transplanted teeth in function and one failed transplanted tooth (R$\frac{4}{4}$, root-end resected left mandibular second premolar) attended the follow-up visit (Fig. 1). On average, the 27 patients are seen 3.4 years (1–16.1 years; Table 1) following autotransplantation.

In the cohort of unmodified teeth, two (a left maxillary first premolar with R$\frac{3}{4}$ and a right mandibular second premolar with R$\frac{3}{4}$) required a root canal treatment 4 and 17 months after transplantation. In the cohort of root-end resected teeth, the need for a root canal treatment came up in one case (a right mandibular second molar) 4 months after surgery.

**Fig. 4:**
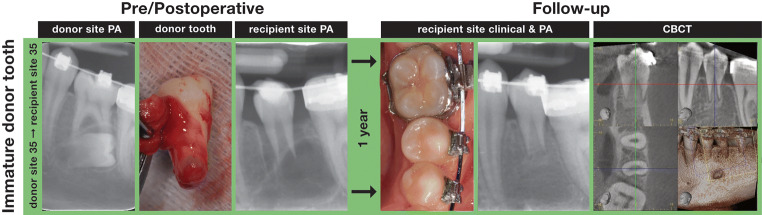
Ingrowth of bone-like tissue into the former pulp chamber is visible in the CBCT of a transplanted lower left second premolar one year after surgery. A “periodontal ligament-like layer” connects this tissue to the dentin walls facing the chamber

PAs were taken in all patients undergoing the follow-up examination (29/29 transplants). The resulting 2D radiographic parameters are presented in Table 3. Postoperative root growth is present in 10 of 23 unmodified teeth, seven of which seemed to achieve the expected root length (Table 1). Interestingly, three teeth with root formation stage of R$\frac{1}{4}$ and one tooth with root formation stage of R$\frac{2}{4}$ show ingrowth of bone-like tissue to the pulp chamber with formation of a periodontal space to the pulpal dentin interface (Fig. 4). Eight of the 29 PAs were deemed inconclusive by the PI regarding root resorptions (*n* = 8) or width of the periodontal ligament space (*n* = 1) and were discussed between the examiners to reach an agreement.

**Table 3:** Radiological findings at the follow-up on PAs for all examined transplants

	PA (29 of 29 teeth)				PA vs. CBCT (20 of 29 teeth)							
	Without root-end resection (*n* = 21)		With root-end resection (*n* = 8)		Without root-end resection (*n* = 15)				With root-end resection (*n* = 5)			
	PA (*n* = 21)		PA (*n* = 8)		PA (*n* = 15)		CBCT (*n* = 15)		PA (n = 5)		CBCT (*n* = 5)	
Pulp canal obliteration	15	71.4%	3	37.5%	12	80 %	12	80%	3	60%	3	60%
Ingrowth of bone-like tissue to the pulp chamber	4	19 %	0	0 %	3	20 %	3	20 %	0	0%	0	0%
Continuous periodontal space	21	100%	6	75%	15	100%	14	93.3%	5	100%	3	60%
Periapical radiolucency	1	4.8 %	1	12.5%	0	0%	0	0%	1	20%	1	20%
Periradicular radiolucency	1	4.8%	0	0%	0	0%	0	0%	0	0%	1	20%
Intraradicular radiolucency (including cervical area)	0	0%	2	25%	0	0%	2	13.3%	1	20%	1	20%

For patients who agreed to undergo an additional CBCT examination, the findings of both 2D and 3D image modalities are compared. The numbers represent the quantity of transplants/teeth showing the specific finding

Nineteen patients with 20 transplants agreed to undergo an additional CBCT examination. The 3D radiographic parameters for all the surviving transplants are presented in Table 3. Surprisingly, CBCT revealed intraradicular radiolucency at the level of the CEJ in two teeth and an interruption of the periodontal space with replacement resorption in three teeth, which was not visible in PAs. Four of the 20 transplants presented with insufficient radiographic evidence for the buccal or oral alveolar bone walls. Three of these had been fully impacted canines, transplanted to their intended position, whereas one was a third molar transplanted to a premolar site. Nine of the 20 CBCTs were deemed inconclusive by the PI regarding width of the periodontal ligament space (*n* = 4), root resorptions (*n* = 3), or the presence of buccal bone walls (*n* = 2) and were discussed between the examiners to reach an agreement. When compared to PA, CBCT yielded additional information for the diagnosis of root resorptions and missing buccal/oral bone.

### Success analysis

Five of the unmodified teeth (21.7%) showed clinical and/or radiographic findings: two were in an infraposition, one presented a perio-endo lesion and two an invasive cervical resorption, which was detectable only on the CBCT scan. Interestingly, five out of seven transplanted molars developed postoperative findings or failed, whereas this was the case only in two of 15 premolars. The single canine in this group exhibited no abnormality. Details are shown in Table 1.

Four of the root-end resected teeth (44.4%) showed postoperative findings, clinically and/or radiographically: the only transplant with preoperative signs of hypercementosis presented with a perio-endo lesion and all three multi-rooted teeth (FDI 18, 46, and 47) showed missing signs of ongoing pulp canal obliteration of which two additionally had increased periodontal pocket depths (8-9 mm). Interestingly, three out of four transplanted molars developed postoperative findings or failed, whereas this was the case in only one of two premolars and one of three canines, i.e., the one with hypercementosis. Details are shown in Table 1.

Finally, the overall success probability after a median follow-up of 3.4 years was 62.5% (20 of 32 transplants) or 69.6% (16 of 23) for transplantations of unmodified teeth after a median observation period of 4.8 years and 44.4% (4 of 9) for root-end resected teeth after a median observation period of 2.5 years.

## Discussion

The purpose of this study was to analyze survival and success rates and related influencing variables of immature and mature autotransplanted teeth in one single center. Upon phone inquiry, survival rates of 92% for immature transplants and 90% of mature transplants with root-end resection were found. Potentially adverse clinical and/or radiographic findings and extracted transplants were found more frequently in the group of mature (55.6%) than immature transplants (30.4%) and for molars (72.7%) compared to premolars (17.6%) or canines (25%). To the best of our knowledge, this is the first clinical study comparing a cohort of transplanted teeth with root-end resection to one without resection.

High survival rates of 90–100% after transplantation have been reported particularly for immature premolars [2, 3, 11, 15, 17, 30]. They have been reported with more favorable outcomes thanks to their root anatomy that facilitates a transplantation without harming the root surface, if compared to any other tooth type or to mature teeth. Furthermore, their frequently wide and single apical foramen promotes revascularization. Expanding treatment indication to other types of teeth such as molars or mature donor teeth results in lower survival rates of 68.2–98% [3, 8, 15, 31–36] due to complex root anatomy or damage of the root surface during tooth harvesting. Moreover, the narrow and frequently multiple apical foramina of, for example, molars impair revascularization [3, 12, 14]. Although only 11 of all teeth assessed in the follow-up visit were molars, eight of them (72.7%) showed postoperative findings, whereas this was the case for only three premolars (17.6%) and one canine (25%).

Transplanted immature teeth are associated with high revascularization rates and promising treatment outcomes, whereas revascularization is rarely seen in mature donor teeth. A positive association between failure and maturity of the transplant could be documented in a meta-analysis, where teeth transplanted with an open apex had 70% less likelihood for subsequent extraction if compared to teeth with a closed apex [7]. Moreover, immature transplants developed less ankylosis or root resorption combined with pulp necrosis. In the present study, 30.4% of the immature transplants developed postoperative findings such as infrapositions (*n* = 2), invasive cervical resorptions (*n* = 2) or apico-marginal lesion (*n* = 1), or failed (*n* = 2). In contrast, 55.6% of the mature transplants developed postoperative findings such as replacement root resorptions (*n* = 3), apico-marginal lesion (*n* = 1), or failed (*n* = 1). Root resorptions led to the extraction of three teeth: one immature molar and premolar and one root-end resected mature premolar. In total, three transplants (two immature, one mature) required subsequent root canal treatment (RCT).

Revascularization rates of the pulp after tooth transplantation appear to correlate with the root formation stage at surgery, due to the width of the apical foramen [3, 7, 14]. Additionally, the influence of the patients’ age at surgery must be discussed, as the proliferation of pulpal stem cells decreases in older patients [37]. Therefore, less capacity for pulp regeneration is anticipated. Upon follow-up, 90.5% of immature transplants did not show a periapical radiolucency and did not require a RCT. This is corroborated by signs of pulp canal obliteration (71.4%) or bony ingrowth to the pulp chamber (19%). The latter appeared in four teeth transplanted at a very early stage of development and was always associated with discontinuation of the root growth. Although similar findings in teeth with R$\frac{2}{4}$ and R$\frac{3}{4}$ have been documented earlier [12], pathogenesis and influencing factors remain uninvestigated. Increasing the diameter of the foramen via intraoperative apical root-end resection was postulated as a method for enhancing pulpal revascularization and healing rates in mature transplants. This was shown to be effective in dog studies [24–26, 38], where necrotic pulp tissue was replaced by well-vascularized and cell-rich connective tissue within 90 days in two-thirds of the cases. Three recent case reports proved the feasibility of this approach for single-rooted teeth scheduled for autotransplantation and showed promising initial outcomes [21–23].

In the present study, 6 of the 9 mature transplants that underwent root-end resection and qualified for the success analysis did not develop endodontic complications clinically and/or radiographically, whereas three developed apical lesions. Out of these, one was associated with ankylosis and an invasive cervical resorption that required extraction, one healed after RCT, and one occurred in an upper canine with hypercementosis but the patient refused to undergo RCT. Moreover, five of the nine reexamined mature transplants showed signs of obliteration as unequivocal evidence of pulpal healing, including the extracted tooth. Two of these five transplants with obliteration additionally showed sensitivity upon CO_2_ testing. Mature donor teeth transplanted without root-end resection show no pulp canal obliteration in the existing literature [12]. Another clinical study documented revascularization in 15% combining electric pulp sensibility testing and/or radiographic signs of obliteration [3]. In conclusion, revascularization of mature transplants seemed enhanced by root-end resection and could be radiologically demonstrated in 55.6% of the cases in the present study. At a closer look, all teeth with more than one root canal and the only tooth with hypercementosis showed no pulp canal obliteration or were extracted, thus accounting for 4 of the 5 non-successful transplants. In other words, 100% of the mature, resected autotransplants with a single root canal and uncomplicated root morphology underwent postoperative pulp canal obliteration. Additional benefits of this approach are the easier preparation of the recipient site, which might be helpful with regard to the safety of neighboring critical anatomical structures. Finally, this results also in a shortening of the surgical time.

To the best of the authors’ knowledge, this is the first study investigating the relevance of CBCT in the follow-up of autotransplanted teeth. When comparing the findings of periapical radiographs and CBCT, most of the replacement and cervical root resorptions detected in this study are not visible using 2D imaging (Table 3). This is corroborated by existing research, where CBCT yielded higher sensitivity than intraoral radiographs in the diagnosis of external root resorptions [39, 40]. However, findings deemed inconclusive by the PI were found more frequently in the assessment of CBCTs than of PAs. Mostly, they were related to the assessment of the periodontal ligament space (physiologic vs. pathologic width). This might be associated with a limited image quality of the CBCTs due to the low-dose protocol used, which may have had an impact especially on the visibility of very fine structures surrounding teeth [41].

A missing buccal or oral bone wall was found in CBCTs of 4 clinically successful transplants. All of these teeth were associated with a narrow alveolar ridge at the recipient site, and all except one were in the anterior maxilla or mandible. This finding is well in line with an early animal study on autotransplantation, showing no regeneration of the alveolar bone in the defect area, i.e., where root surfaces were exposed to soft tissue [42]. In this study, the desmodontal fibers of the intact root surface connected directly to the adjacent soft tissue without interposition of bone, as it might have happened in the present study around teeth transplanted without sufficient alveolar bone width. Nevertheless, a missing buccal bone plate has been postulated as a risk factor for the loss of the transplant in two clinical studies [32, 43]. On the other hand, CBCT shows low sensitivity in diagnosing a thin alveolar bone wall, and thus, the present findings should be interpreted with caution [44]. In other words, an alveolar bone wall may have been present but could have been too thin to be detected in CBCT especially when using a low-dose protocol. In comparison to PA, CBCT delivered additional information in only 5 of the 20 cases, i.e., 5 root resorptions without need for treatment. Thus, in light of the limited clinical relevance of such additional diagnostic information, indication for postoperative CBCT follow-up on a routine base may be critically questioned.

Limitations of the present investigation include the retrospective study design, the small sample size, varying tooth types, and heterogeneities observed in the treatment protocols chosen. In particular, indication-dependent splinting protocols and inhomogeneous use of EMD were found. The limited number of included cases may negatively affect the significance of the analysis, for example, when comparing the groups mature/immature or when assessing the influence of potential predictors. However, this is the first clinical study evaluating the effect of extraoral root-end resection in mature autotransplanted teeth, including an appraisal of the potential role and value of 3D imaging in the follow-up of transplanted teeth. Further research focusing on possible factors influencing the outcome of larger patient cohorts with mature transplants, preferably with perioperative RCT as a control group, is needed.

## Conclusions

Immature autotransplanted teeth yielded a satisfying average midterm survival rate of 92%. Extraoral root-end resection appears to be a potential approach for inclusion also of mature teeth as candidates for autotransplantation after careful case selection. This technique shows promising outcomes for transplants with a single root canal and uncomplicated root morphology. Regarding the diagnosis of root resorptions, CBCT may be helpful in selected cases but cannot be recommended as a routine follow-up imaging modality.
